# Harmonization Study Between LC‐MS/MS and Diasorin RIA for Measurement of 25‐Hydroxyvitamin D Concentrations in a Large Population Survey

**DOI:** 10.1002/jcla.22049

**Published:** 2016-09-06

**Authors:** Diane J. Berry, John Dutton, William D. Fraser, Marjo‐Riitta Järvelin, Elina Hyppönen

**Affiliations:** ^1^ Population, Policy and Practice UCL Institute of Child Health London UK; ^2^ Department of Clinical Biochemistry Royal Liverpool University Hospital Liverpool UK; ^3^ Norwich Medical School University of East Anglia Norwich Research Park Norwich UK; ^4^ Department of Epidemiology and Biostatistics MRC Health Protection Agency (HPA) Centre for Environment and Health School of Public Health Imperial College London London UK; ^5^ Unit of Primary Care Oulu University Hospital Oulu Finland; ^6^ Department of Children, Young People and Families National Institute for Health and Welfare Oulu Finland; ^7^ Biocenter Oulu Institute of Health Sciences University of Oulu Oulu Finland; ^8^ Centre for Population Health Research School of Health Sciences and Sansom Institute of Health Research University of South Australia Adelaide Australia; ^9^ South Australian Health and Medical Research Institute Adelaide Australia

**Keywords:** 25(OH)D, assay, limits of agreement, population study, reliability, replication, vitamin D

## Abstract

**Background:**

Population‐based research on vitamin D has increased dramatically in recent years. Such studies are typically reliant on assay procedures to measure reliable and comparable levels of 25‐hydroxyvitamin D [25(OH)D] concentrations.

**Methods:**

Concentrations of 25(OH)D_3_ and 25(OH)D_2_ were measured using LC‐MS/MS in 5,915 participants (aged 31 years) of Northern Finland Birth Cohort 1966. Blood samples were assayed in batches over a course of 18 months. As anomalies were present in the measurements, 200 samples were reassayed using Diasorin RIA. Agreement between measurements was assessed by Passing–Bablok regression and limits of agreement (LoA). To harmonize LC‐MS/MS with Diasorin RIA measurements, formulae were derived from the LoA.

**Results:**

Concentrations measured by LC‐MS/MS were much higher than those measured by Diasorin RIA, with a mean difference of 12.9 ng/ml. Constant variation was evident between batch measurements after log transformation. Statistical formula was applied separately for each batch of LC‐MS/MS measurements, enabling us to remove both the constant and proportional bias that was evident prior to the transformation.

**Conclusion:**

Despite the introduction of schemes/programs to improve accuracy of assays to measure 25(OH)D, significant differences can still happen. In these instances, methods to harmonize measurements based on a relatively small number of replicates can be successfully applied to establish confidence and to enable between‐study comparisons.

## Introduction

Epidemiological research on vitamin D has increased dramatically in recent years as the role of vitamin D in health is suspected to be broad and far reaching 1. However, there are significant discrepancies between procedures used to estimate vitamin D status 2, 3. Vitamin D is a prohormone and is mostly obtained through skin synthesis from UVB exposure, and as such shows strong seasonal trends 4, 5, 6. Vitamin D can be obtained in two isoforms, and while UVB exposure‐related synthesis will always lead to formation of cholecalciferol (D_3_) 6, in diet or dietary supplements, vitamin D can also exist as egrocalciferol (D_2_) 7. Vitamin D status can be estimated by measuring total circulating 25‐hydroxyvitamin D (25(OH)D) concentrations, which is the result of the combination of hydroxylated forms of vitamin D_2_ and D_3_ 8.

There are a variety of assay techniques available to measure 25(OH)D concentrations and some have the ability to measure the two forms 25(OH)D_2_ and 25(OH)D_3_ 2. Reliable measures across techniques are essential to have confidence in the concentrations for clinical practice and to allow comparison across research studies, such as the prevalence rates of vitamin D deficiency/sufficiency. The International Vitamin D External Quality Assessment Scheme (DEQAS) was established in 1989 with the aim to “monitor the performance of individual laboratories” 9. Participating laboratories receive a certificate if 80% of their results from the five quarterly samples are within 30% of the all‐laboratory trimmed mean (ALTM) 9. The Vitamin D Standardization Program (VDSP) was recently established to promote the need for accurate and comparable 25(OH)D measurements and has a focus on large national surveys 10. A protocol is being developed by the VDSP to standardize 25(OH)D concentrations from past surveys, which can entail reanalyzing banked sera in a certified laboratory and developing an equation to harmonize past results with recent sera results.

In 2008, we measured 25(OH)D_2_ and 25(OH)D_3_ in 5,915 participants of the 1966 Northern Finland Birth Cohort (NFBC1966) 11 using a liquid chromatography‐mass spectrometry technique (LC‐MS/MS). LC‐MS/MS is an automated system with high throughput and is able to measure separately 25(OH)D_2_ and 25(OH)D_3_ 12. It is an ideal procedure for a population study and accordingly is becoming frequently used 13. However, after receiving the results we suspected that there were some measurement anomalies in the results obtained by LC‐MS/MS on NFBC1966 samples, as suggested by the very high average 25(OH)D concentrations in this Nordic population. To validate the measurements we systematically selected a subsample of 200 participants and reanalyzed these samples using a common kit method, Diasorin RIA 14. We then assessed the limits of agreement (LoA) between the two methods and the DEQAS results for the laboratory used. On the basis of the limits we sought to statistically harmonize the LC‐MS/MS measures to Diasorin RIA and justify the transformation through seasonal trends and vitamin D dietary supplement information.

## Methods

The NFBC1966 surveyed births in the year 1966 in Finland's northern provinces of Oulu and Lapland 15. The original cohort included over 12,000 deliveries and the survivors have been followed to adulthood. In 1997, cohort members residing in Finland with known addresses (*n* = 11,541) were invited to participate in the postal questionnaire. A subsequent invitation to a clinical examination was sent out to the participants of the postal questionnaire residing in the regions of Oulu and Lapland, and in the capital city Helsinki. Of those who responded to the postal questionnaire, 70% (*n* = 5,915) consented to a medical examination and had a stored blood sample.

In 2008, the frozen serum samples were defrosted and the 25(OH)D_2_ and 25(OH)D_3_ concentrations were measured using LC‐MS/MS. Data were received at four stages, the first in March 2008 and the last in August 2009, and the batches were correlated with original date of storage in the Finnish laboratory in 1997. In total, 5,608 participants had measurements of 25(OH)D concentrations. From each of the four batches we systematically selected 50 samples over a range of the 25(OH)D_3_ and 25(OH)D_2_ concentrations for replication with the Diasorin (Stillwater, MN) RIA (a radioimmunoassay with a ^125^I‐labeled tracer). During the period of study sample assessment (March 2008–August 2009), the laboratory took part in five rounds of DEQAS evaluations, returning results using LC‐MS/MS for all five samples which were sent each time. Internal Quality Assurance samples were included at the front and back of each assay. The materials, solution, preparation and LC‐MS/MS system used are detailed below.

### Materials and Chemicals

25(OH) Vitamin D_2_ (25(OH)D_2_) (cat no. 17937) and 25(OH) Vitamin D_3_ (25(OH)D_3_) (cat no. H4014) were obtained from Sigma Chemical Company (Gillingham, Dorset, UK) and the internal standard of 26,27 hexadeuterium 25(OH)D_3_ was purchased from Synthetica AS, Oslo, Norway. HPLC grade methanol, propan‐2‐ol, acetonitrile and tetrahydrofuran were obtained from VWR. Ultrapure deionized water (>18.2 MΩ/cm) was obtained from a Millipore (Watford, Hertfordshire, UK) AFS 50 EDI unit. Isolute C18(EC) 200 mg 3 ml reservoirs were purchased from Kinesis Ltd (St Neots, Cambridgeshire, UK). A stream of nitrogen was provided by a Peak Scientific nitrogen generator, at 40°C in a Techne Dryblock DB3 (Sigma Chemical Company).

### Stock Solutions, Calibration Standard Solutions and Control Samples

Individual calibrator stock solutions (50 mg/l) of each metabolite were prepared in ethanol, and their concentrations checked using a Cary 1E double beam spectrophotometer (Varian, Walton on Thames, Surrey, UK), using molar absorptivities at 264 nm (1 cm path length) of 19,400 and 18,300 for 25(OH)D_2_ and 25(OH)D_3_, respectively. From these primary stock calibrators, an intermediate combined calibrator was prepared by diluting 100 μl of stock 25(OH)D_2_ and 200 μl of stock 25(OH)D_3_ in 25 ml of 0.9% saline. An eight‐point working calibration curve was then prepared in 22% bovine albumin (Lorne Laboratories, Lower Earley, Berkshire, UK), to cover the range up to 59.7 ng/ml for 25(OH)D_2_ and 183 ng/ml for 25(OH)D_3_. Endogenous concentrations of 25(OH)D_2_ and 25(OH)D_3_ present in the bovine albumin were electronically compensated by the Quanlynx software (Waters, Elstree, Hertfordshire, UK). Working standards are prepared freshly with each batch. Internal standard was prepared by dissolving 5 mg d6 25(OH)D_3_ in 200 ml methanol/propan‐2‐ol. A further dilution of 1:1,000 in methanol/propan‐2‐ol (9:1 v/v) was made to obtain a working concentration of 125 ng/ml.

### Sample Preparation

Samples were prepared for analysis by thawing, mixing and recentrifuging, to remove any fibrin debris. One milliliter of sample, calibrator, or quality control material was pipetted into 10 ml screw top glass tubes. To this was added 1 ml of working internal standard. Contents were vortex mixed thoroughly for at least 30 s. Then 2 ml of acetonitrile was added and again vortex mixed for at least 30 s. Tubes were left at 4°C for 1 h before centrifugation at 2,000 × *g* for 10 min. Supernatants were transferred to 13 × 100 mm disposable glass tubes and placed in the sample zone of the Gilson ASPEC XL4 (Anachem, Luton, UK) and protected from natural sunlight. The XL4 processed four samples simultaneously in approximately 12 min. The instrument sequentially conditioned the C18 solid‐phase extraction cartridges (SPE) in the disposable enrichment cartridge (DEC) zone with 3 ml of methanol followed by 3 ml of water, to firstly activate and then condition the SPE prior to sample introduction. Three milliliters of sample extract was then introduced onto the SPE followed by a wash with 2.5 ml of 50:50 methanol:water. Elution of 25(OH)D_2_ and 25(OH)D_3_ was achieved with 3.0 ml of 10% tetrahydrofuran in acetonitrile. The eluate was dried at 40°C under a stream of nitrogen. Dry extract was reconstituted in 100 μl of 75% methanol/water and vortex mixed for 10 s, prior to transferring to a 150 μl conical microvial and loading into the Aquity sample racks. Extracts were stable for up to 1 week at 4°C.

### LC‐MS/MS System

The integrated HPLC system used to separate the peaks of interest was a Waters Acquity Ultra Performance Liquid Chromatography system (Elstree, Hertfordshire, UK). Chromatographic separation was achieved using a Waters SunFire C18 (Elstree, Hertfordshire, UK) (3.5 μm 100 mm × 2.1 mm id) analytical column fitted with a guard column. Column life was improved by fitting a 10 mm C18 guard column. Column temperature was maintained at 40°C. A Waters Premier XE (Elstree, Hertfordshire, UK) with a Z spray source, in ESI positive MRM mode with a source temperature maintained at 130°C, desolvation temperature at 250°C, gas flow of 950 l/h, and argon collision gas set at 0.3 ml/min was used for mass detection. QuanLynx software was used to calculate relative retention time for peak identification and peak area ratios with internal standardization for quantitation. Mobile phase A contained 2 mmol/l ammonium acetate (VWR) in 0.1% formic acid (Fluka Chemical Company; Sigma‐Aldrich). Mobile phase B contained 2 mmol/L ammonium acetate in methanol containing 0.1% formic acid. A binary step gradient was used to clean the column of any late eluting peaks. Elution of the vitamins was achieved using 84% B at 0.4 ml/min for 3.5 min then switching to 100% B for a further minute before returning to 84% B. Injection interval was set to 5.5 min, allowing 1 min for re‐equilibration of the column before the next injection. Solvent divert was used to allow data acquisition to take place between 1.2 and 3.5 min. Eluent was then introduced into a Waters Premier XE (Elstree, Hertfordshire, UK) tandem mass‐spectrometer fitted with an electrospray ionization source. The sample tray area was maintained at 15°C. Quantitation MRM transitions were 401.1 > 383.2, 413.2 > 395.2, and 407.1 > 107.1 for 25(OH)D_3_, 25(OH)D_2_, and d6 25(OH)D_3_, respectively.

Diasorin RIA was obtained from the manufacturer (Diasorin) and the procedure for use was followed as described in the pack insert.

### Vitamin D Dietary Supplements

The postal questionnaire sent to participants of the 31‐year survey asked “How often do you use the following medication?… Vitamins or trace elements (1) Not at all, (2) Sometimes, (3) Regularly or continually…” and to list all medicines taken at present with strength and dose. In the coding of the questionnaire each of the participants' medicine was given with an associated Anatomical Therapeutic Chemical (ATC) code 16. Vitamins were coded with A11 “Vitamins” or A12 “Mineral Supplements.” For all supplements that were listed under the two ATC codes, a Google search was performed to see if they contained a vitamin D_2_ or D_3_ compound and quantity. If this was not clear from the distributer or company website, emails were sent asking for information. Two more subsequent emails were sent to the same email address and any other contact emails found on the websites if a reply was not received within a few weeks. The medicine listing was double checked for misspellings and missclassified ATC codes.

### Statistical Methods

We assessed the agreement between the methods and the DEQAS ALTM using Passing–Bablok regression, which does not make distributional assumptions regarding the data 17. Further investigation was done by assessing the LoA as outlined by Bland and Altman 18. The LoA of the measurements were interrogated by plotting the difference between the two methods of measurement (as denoted by *D*_*i*_ = *y*_1*i*_−*y*_2*i*_, where *y*_1_ and *y*_2_ are the respective methods for *i *=* *1…*N* pairs of measurements) against the average of the measurements [as denoted by *A*_*i*_ = (*y*_1*i*_−*y*_2*i*_)/2]. To compensate for increases in variability over increasing magnitude, the natural log transformation was used. The log‐transformed measures were reinterrogated using LoA to identify potential outliers, and this was also stratified by the stages (batches) that the measures were received from the laboratory.

Evidence for non‐constant difference was assessed by using linear regression of the difference between measures adjusted for the mean of the measures (Eqn. (1)).

(1) $$D_{i}=\lambda +\mu A_{i}+\varepsilon_{i},\text{where}\;\varepsilon_{i}∼N\left(0,\tau^{2}\right)$$

The absolute residuals from the model were used in a linear regression model adjusted for the mean of the measures to assess for evidence of non‐constant variance 18. As a non‐constant difference was evident for the mean but not for the variance, the LoA were converted into a prediction formula to harmonize the LC‐MS/MS measurements to the Diasorin RIA method 19. The prediction formula from one method to another (Eqn. (2)) is calculated from the coefficients estimated in the regression with differences in means (Eqn. (3)) 19.

(2) $$\;\;\;\;y_{20}=\alpha_{2/1}+\beta_{2/1}y_{10}\pm 2\times \sigma_{2/1}$$

(3) $$\begin{array}{c} \alpha_{2/1}=\frac{-\lambda }{\left(1+\mu /2\right)}\\ \beta_{2/1}=\frac{\left(1-\mu /2\right)}{\left(1+\mu /2\right)}\\ \sigma_{2/1}=\frac{\tau }{\left(1+\mu /2\right)} \end{array}$$

The prediction formulae were calculated for the sample and again individually by the batches (Eqn. (4)).

(4) $$\begin{array}{cc} & \text{A‐Jun}\;2008:\;\;y_{\mathrm{RIA}}.9pt=0.59+0.70\;y_{\mathrm{LC}-\mathrm{MS}/\mathrm{MS}}\pm 2\;\left(0.30\right)\\ & \text{B‐Nov}\;2008:\;y_{\mathrm{RIA}}.2pt=1.15+0.60\;y_{\mathrm{LC}-\mathrm{MS}/\mathrm{MS}}\pm 2\;\left(0.24\right)\\ & \text{C‐Mar}\;2009:\;y_{\mathrm{RIA}}.7pt=1.37+0.50\;y_{\mathrm{LC}-\mathrm{MS}/\mathrm{MS}}\pm 2\;\left(0.28\right)4\\ & \text{D‐Apr}\;2009:\;y_{\mathrm{RIA}}=0.34+0.86\;y_{\mathrm{LC}-\mathrm{MS}/\mathrm{MS}}\pm 2\;\left(0.28\right) \end{array}$$

Agreement between the harmonized and Diasorin RIA measures was assessed using Passing–Bablok regression. Finally, to compare the effect of harmonization, the monthly variation was inspected and the effect of vitamin D diet supplement on total 25(OH)D was examined by regressing on the log‐transformed LC‐MS/MS, Diasorin RIA, and harmonized LC‐MS/MS measures adjusting for season. The analysis was done using the software Analyse it (version 2.20) 20 and Stata (version 12) 21.

## Results

**Figure 1:**
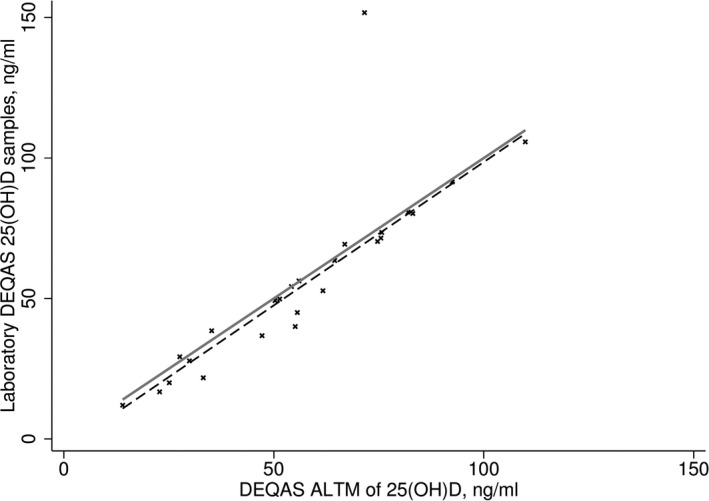
25‐hydroxyvitamin D concentrations as measured from the Vitamin D External Quality Assessment Scheme (DEQAS) samples in the study laboratory compared to the mean from all participating laboratories (DEQAS all‐laboratory trimmed mean [ALTM]). Passing–Bablok regression (dashed line) compared to identity (solid line).

One obvious outlier was identified examining results from Passing–Bablok regression comparing the laboratory submitted samples to DEQAS and the DEQAS ALTM. The values from all other DEQAS samples submitted from the laboratory using the LC‐MS/MS were very close to the ALTM. Excluding the one outlying observation had only a minor difference to the relationship and we found no evidence of constant or proportional bias (*P*‐value>0.1, Fig. 1). The performance of the LC‐MS/MS based on internal standards was fair with %CV for 25(OH)_2_D_2_ 12.1% at 9.2 nmol/l, 9.1% at 24.8 nmol/l, 4.8% at 52.8 nmol/l, and 3.9% at 158.5 nmol/l. For 25(OH)_2_D_3_ %CV was 15.5% at 6.1 nmol/l, 5.8% at 19.6 nmol/l, 4.3% at 58.1 nmol/l, and 3.6% at 124.5 nmol/l.

**Figure 2:**
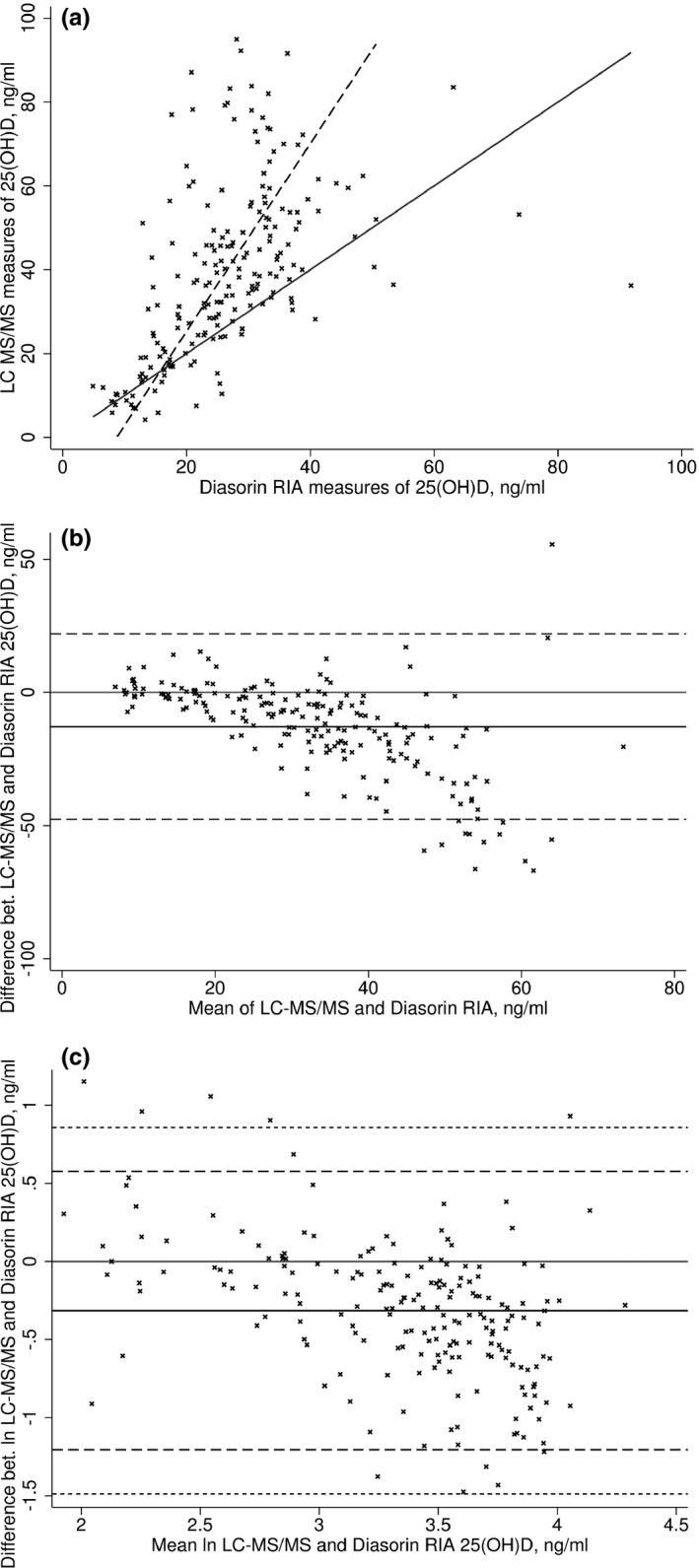
Positive bias in 25‐hydroxyvitamin D concentrations measured by liquid chromatography‐mass spectrometry technique (LC‐MS/MS) compared to Diasorin RIA. (A) Passing–Bablok regression (dashed line) compared to identity (solid line). (B) Mean difference in concentrations (ng/ml, solid line) with limits of agreement (95% LoA). (C) Mean difference after log transformation with limits of agreement (95% LoA dashed line; 99% LoA short dashed line).

Comparing the measures in the replicated sample from LC‐MS/MS and Diasorin RIA, we observed a constant and proportional bias with a mean difference of 12.9 ng/ml (*P*‐value ≤ 0.0001) (Fig. 2A). A non‐constant difference was seen in the untransformed and natural log‐transformed measures (*P*‐values ≤ 0.0001 for both, Fig. 2B, C). Five observations were seen outside of the upper 99% LoA (Fig. 2C) and these observations were excluded from harmonization equations for the sample.

**Figure 3:**
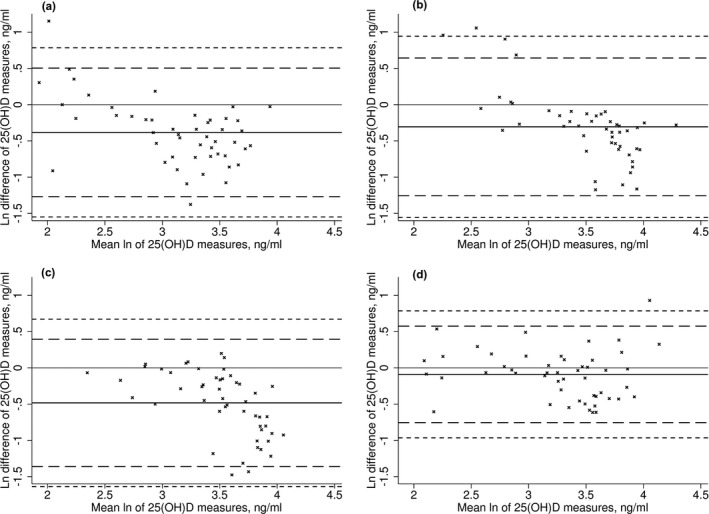
The difference in the log‐transformed 25‐hydroxyvitamin D concentrations measured by LC‐MS/MS and Diasorin RIA by batch (A‐Jun 2008, B‐Nov/Dec 2008, C‐Mar 2009, D‐Apr 2009). The solid black line is the mean log difference, dashed line 95% LoA, and short dashed line 99% LoA.

Investigating the sample by the batches that they were received in from the laboratory, we again saw the five outlying observations and excluded a further additional observation from batch B (Fig. 3B) from the calculations with the batches. We found there was no evidence for non‐constant variance in the natural log‐transformed measures across the sample and when analyzed as separate batches (*P*‐value ≥0.11 for individual batches and sample). Given, the constant variance we were able to calculate the prediction formulae to harmonize the LC‐MS/MS measures by extension of the LoA.

**Figure 4:**
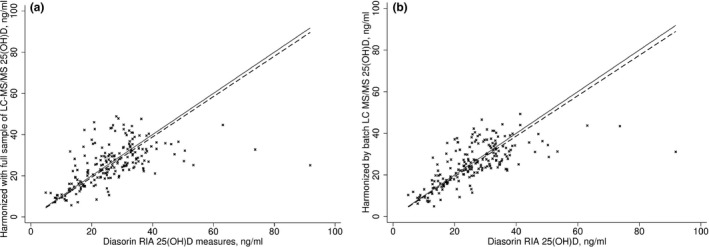
Linear association in 25‐hydroxyvitamin D concentrations measured by liquid chromatography‐mass spectrometry technique (LC‐MS/MS) and Diasorin RIA after statistical harmonization. The solid black line is the relationship from Passing–Bablok regression and the dashed line is the identity. (A) LC‐MS/MS measures harmonized by single formula; (B) LC‐MS/MS measures harmonized by batch‐specific formulae.

**Figure 5:**
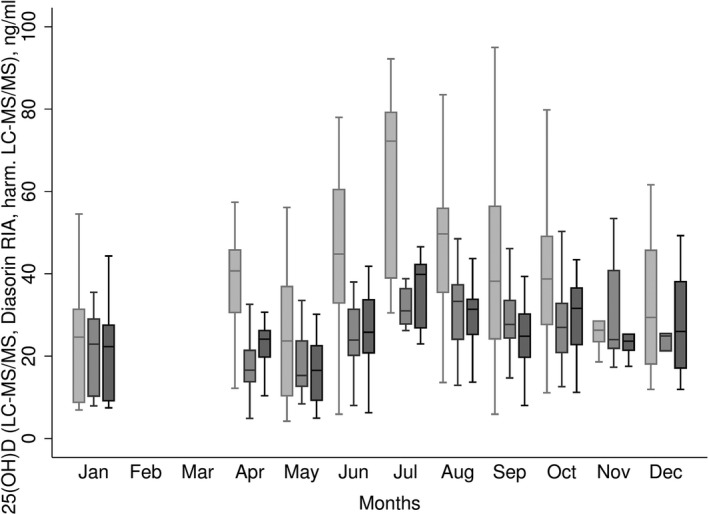
Monthly variation in 25(OH)D concentrations for liquid chromatography‐mass spectrometry technique (LC‐MS/MS) (untransformed, light grey), Diasorin RIA (mid‐grey), and harmonized LC‐MS/MS (dark grey) measures. Shown as the mean with 25^th^ and 75^th^ percentiles (box), range given by error bars.

**Figure 6:**
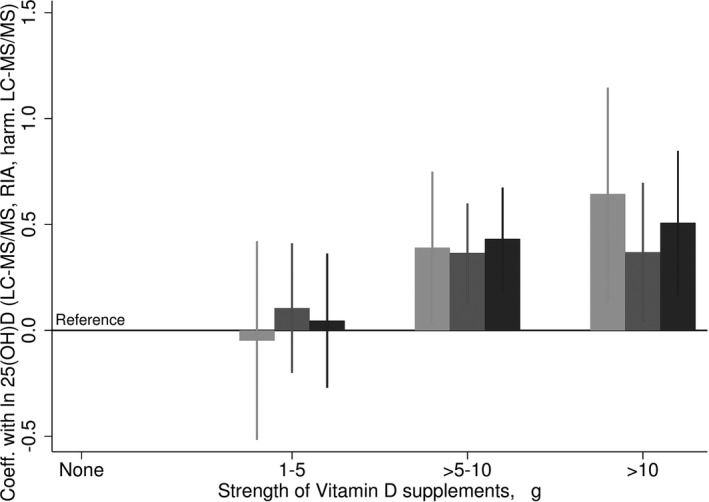
The association of vitamin D supplementation with 25‐hydroxyvitamin D concentrations measured by liquid chromatography‐mass spectrometry technique (LC‐MS/MS) (untransformed, light grey), Diasorin RIA (mid‐grey) and harmonized LC‐MS/MS (dark grey) from a linear regression model adjusted for season. Ninety‐five percent confidence intervals given by error bars.

After harmonizing the LC‐MS/MS measures using a prediction formula for the sample and individual formulae for the batches (Eqn. 4), the constant and proportional bias in concentrations measured by LC‐MS/MS compared to Diasorin RIA that was evident before transformation was removed (Fig. 4). The best agreement between the assays methods was obtained by carrying out the harmonization in batches. Furthermore, the seasonal pattern observed for the harmonized measures demonstrated a clearer pattern than the non‐harmonized LC‐MS/MS measures, with the pattern appearing visually similar to the Diasorin RIA measures (Fig. 5). The median values and prevalence of 25(OH)D above 40 ng/ml in the harmonized 25(OH)D concentrations were similar with concentrations measured by Diasorin RIA (6.0% and 6.6%, respectively, Table 1), and notably reduced from the 45.5% before transformation. Consistent with the lower mean and narrower range in the values, vitamin D supplementation was associated with slightly smaller increases in 25(OH) D concentrations with the harmonized measures compared with the non‐harmonized measures, however, confidence limits were also somewhat tighter (Fig. 6).

**Table 1:** Description of LC‐MS/MS, Diasorin RIA, and Harmonized LC‐MS/MS 25‐Hydroxyvitamin D Measures, ng/ml in the Replicated Sample (*n* = 200)

	Median (IQR)	Supplements		<10 ng/ml % (*n*)	>40 ng/ml % (*n*)
		Yes Median (IQR)	No Median (IQR)		
LC‐MS/MS					
Total 25(OH)D	37.2 (24.0, 52.0)	41.3 (31.0, 54.0)	36.9 (22.5, 52.0)	6.5 (13)	45.5 (91)
25(OH)D_2_	3.2 (1.6, 10.7)	17.5 (3.3, 24.3)	2.7 (1.4, 6.4)	NA	NA
25(OH)D_3_	28.6 (16.8, 45.5)	21.7 (15.0, 29.4)	30.4 (17.2, 46.6)	NA	NA
Diasorin RIA					
Total 25(OH)D	25.7 (18.6, 32.8)	29.3 (23.2, 34.0)	25.6 (17.6, 32.8)	4.1 (8)	6.6 (13)
Harmonized LC‐MS/MS					
Total 25(OH)D	26.2 (18.8, 32.8)	27.4 (25.2, 36.8)	24.9 (18.0, 32.1)	6.5 (13)	6.0 (12)

## Discussion

In our study, we were able to adjust 25(OH)D concentrations measured by LC‐MS/MS to be in line with a single replicate measured from the Diasorin RIA using an extension of LoA. The logic we have used to achieve harmonization is similar to a protocol that is being developed by VDEP to harmonize 25(OH)D from large studies 10. The harmonization has been based on regression equations that extend the LoA 19, this is also similar to the procedures proposed by the National Center for Environmental Health to address measurement issues related to variability in the Diasorin assay in NHANES surveys 22. By harmonizing the measurements of 25(OH)D, the prevalence rates of 25(OH)D deficiency/sufficiency and effect sizes found with health outcomes in this large population survey can be more readily compared with rates from other studies.

In general, it has been reported that 25(OH)D concentrations measured by Diasorin RIA and LC‐MS/MS procedures are in close agreement (reported correlation 0.91–0.98) 23, 24, 25, 26, 27. Close agreement has been reported in a range of conditions, including studies in participants with relatively high 25(OH)D_2_ concentrations 23, with no detectable 25(OH)D_2_ concentrations 25, with detectable levels of C‐3 epimer of 25(OH)D 27, and with elevated vitamin D binding protein levels 26. However, moderate and possibly non‐linear agreement between the procedures has also been observed 28, 29, illustrating potential for methodological issues with regard to the use of different assays.

For the NFBC1966 measurements of 25(OH)D, it remains unclear why there were anomalies in the measurements from LC‐MS/MS. The blood samples were stored in a freezer for 11 years prior to 25(OH)D measurement, however, some authors have reported that concentrations of 25(OH)D in serum and plasma are unaffected by long‐term storage and iterations of freeze‐thaw cycles 30, 31. However, we are able to discount the possibility of storage‐related interference by unknown metabolites in our samples affecting measures obtained by LC‐MS/MS. There have been concerns about 3‐epi‐25(OH)D_3_ interference in 25(OH)D_3_ measurement by LC‐MS/MS 32. While this could have had some effect on 25(OH)D_3_ measures in our study, it is likely to be of limited importance given the low circulating concentrations of 3‐epi‐25(OH)D_3_ in adults. A potential source of error is the use of in‐house calibrators and internal standardization in the LC‐MS/MS procedure 33. To this end, US agencies and government departments have developed a serum‐based reference material and it is hoped that this will improve assay comparability 2. Since the reporting of DEQAS data in July 2005, the level of recovery 25(OH)D concentrations has improved compared with ALTM, and the proportion of laboratories using LC‐MS/MS has increased at the same time 34, 35. However, the use of ALTM as the standard has its own issues. The ALTM has moved away from the gold standard (gas chromatography‐mass spectrometry) as more methods are used to measure 25(OH)D 35. A passing result by DEQAS does not deem a method as accurate, but as being relatively similar compared with other laboratories that used that method 36, 37.

In our study, we resampled 200 participants with the Diasorin RIA for several reasons. Diasorin RIA has been used extensively in RCTs, clinics, and research studies and has been commercially available for a long time 37. As with LC‐MS/MS it requires a skilled technician to run the assay, however, it has been concluded that in terms of convenience, speed, turnaround, and cost, a well‐known immunoassay is a reasonable choice 38. In some studies, Diasorin RIA assay has been reported to recover up to 100% of 25(OH)D_2_ and 25(OH)D_3_ concentrations 39. Nevertheless, it is recognized that Diasorin RIA assay is not fully exempt from issues of 25(OH)D_2_ recovery 22, 40. As we have demonstrated in this study, a direct comparison of agreement against Diasorin RIA (as an example of an alternative, commonly used assay) is a possible solution when the use other references such as the DEQAS results is impractical, as was previously done by one of the authors 41. However, despite achieving better agreement between the methods by the statistical harmonization of 25(OH)D concentrations, any use of these data for developing population health guidelines should be done with caution.

The prediction equations used to harmonize the methods were based on one measurement (per sample) from each assay. Another option may have been to use prediction equations from Deming regression, but this would have assumed that there was a fixed value for the ratio of variances 19. In an earlier study, it was observed that at low 25(OH)D concentrations, LC‐MS/MS gave higher values than Diasorin RIA, whereas at high levels LC‐MS/MS measures were lower than Diasorin RIA 28. To investigate whether deviations in the agreement between LC‐MS/MS and Diasorin in the NFBC1966 measures were dependent on the concentrations, prediction equations were derived after stratifying the sample by levels of 25(OH)D_2_ and 25(OH)D_3_ (data not shown). However, stratification by level did not improve the prediction equations. Nevertheless, it is possible that information at the high extremes of the 25(OH)D concentrations may have been lost by harmonizing as overall variation has been reduced.

In conclusion, measures of 25(OH)D concentrations in large population surveys are a vital source of information for research and the forming of public health messages. In this study we have illustrated the uses of a statistical procedure that can be used to harmonize the distribution of 25(OH)D concentrations across assays, which will improve the ability to use general cut‐offs to indicate low/high concentrations.
